# Venous thromboembolism after hip arthroscopy: a systematic review of incidence, risk factors, and international guidelines

**DOI:** 10.3389/fsurg.2025.1658428

**Published:** 2025-10-14

**Authors:** Ting Gao, Kai Lv, Hong Zhang, Jian Chen, Xiangde Zhao

**Affiliations:** 1 Nursing Department, Sir Run Run Shaw Hospital, Zhejiang University School of Medicine, Hangzhou, China; 2 Department of Orthopaedic Surgery, Sir Run Run Shaw Hospital, Zhejiang University School of Medicine, Hangzhou, China; 3 Zhejiang Key Laboratory of Mechanism Research and Precision Repair of Orthopaedic Trauma and Aging Diseases, Hangzhou, Zhejiang, China

**Keywords:** arthroscopy, hip, practice guidelines, risk assessment, thromboprophylaxis, venous thromboembolism

## Abstract

**Purpose:**

This review aims to systematically evaluate the incidence, risk factors, and international guideline discrepancies for venous thromboembolism (VTE) following hip arthroscopy (HA).

**Methodology:**

A search of four databases from the inception to April 20, 2025, identified studies reporting VTE outcomes post-HA. Relevant practice guideline recommendations were concurrently analyzed.

**Results:**

Twenty-one studies encompassing 135,377 patients and five clinical guidelines were included. Female patients constituted 91,013 cases (67.2%). The mean patient age was 37.08 years; however, the average follow-up duration was limited to 3.7 months, which may be a study limitation. Pooled incidence rates were: deep vein thrombosis (DVT) 0.441%, pulmonary embolism (PE) 0.216%, and overall VTE 0.656%. The majority of studies were Level IV evidence (57%), with Level III evidence comprising 33%. Identified risk factors for post-HA VTE included obesity, oral contraceptive use, ≥45 years, overweight status, coagulopathy, and arteriovenous anomalies. The reported VTE incidence ranged from 0% to 6.94%. International guidelines vary, but most advocate for risk-stratified thromboprophylaxis.

**Conclusions:**

The incidence of VTE following hip arthroscopy is low. Routine pharmacological thromboprophylaxis may not be necessary for standard-risk patients. However, high-risk individuals warrant personalized prophylaxis regimens, with pharmacological prophylaxis when clinically indicated based on risk assessment.

## Introduction

Since Dr. Michael Burman performed the first hip arthroscopy in 1931 (1), the annual volume of hip arthroscopic procedures has progressively increased, with a marked acceleration in recent years (2). HA helps doctors better diagnose and treat hip conditions such as femoroacetabular impingement, a leading cause of labral tears and early arthritis. With its widespread adoption, complications associated with HA have garnered increasing attention, including femoral neck fractures, avascular necrosis of the femoral head, iatrogenic chondral injury, temporary neuropraxia, DVT, and PE (3). Reported rates of postoperative thrombosis following HA range from 0.2% to 9.5% (4). Despite lower VTE rates compared to joint replacement (5), post-arthroscopic thrombosis has received limited attention. However, the annual performance of tens of thousands of hip arthroscopies ensures that the absolute number of thromboembolic complications is not trivial (6).

A UK study analyzed hip arthroscopy patients through England's National Health Service admissions database, reporting a 0.16% venous thromboembolism (VTE) rate (7). In contrast, a US study using the Mariner Ortho Pearl Database (2015–2021) found a higher incidence of 0.75% post-surgery (8). A recent study leveraging The TriNET Research Network (2003–2024)—which houses one of the largest clinical datasets across North America and Western Europe—documented the highest VTE rate at 1% following these procedures (9). Among untreated proximal DVT cases, approximately 10% to 30% progress to symptomatic PE. However, imaging studies suggest that the proportion of asymptomatic PE may reach 50% (10). The incidence of untreated PE is approximately 5% to 30%, which prolongs hospitalization, increases costs, and necessitates prolonged anticoagulation (>3 months) (10, 11), thereby compromising the minimally invasive advantage of arthroscopic surgery.

Multiple factors have been confirmed as risk factors for thrombosis, including obesity, oral contraceptive use, age ≥45 years, and overweight status (12). However, no detailed guidelines exist for thromboprophylaxis for hip arthroscopy because orthopedic surgeons are not fully aware of the risks. This review aims to examine and compile the existing literature reporting VTE after HA to evaluate the incidence and risk factors for VTE following HA. This review synthesizes incidence, risk factors, and prophylaxis strategies for VTE following HA.

## Methods

### Registration

This systematic review was conducted in accordance with the Preferred Reporting Items for Systematic Reviews and Meta-Analyses (PRISMA) guidelines and prospectively registered with PROSPERO (registration number: CRD420251035449).

### Eligibility criteria

Inclusion criteria comprised (1): studies reporting incidence of VTE, DVT, or PE following HA; (2) investigations evaluating physical or pharmacological VTE prophylaxis strategies post-HA; and (3) clinical guidelines addressing VTE prevention after arthroscopic procedures. Exclusion criteria encompassed animal studies, editorials, commentaries, and studies lacking explicit quantitative VTE outcome data.

### Search strategy

Two independent reviewers systematically searched PubMed, Web of Science, China National Knowledge Infrastructure and Embase databases from inception to April 20, 2025. Search terms included: (‘venous thromboembolism’ OR ‘VTE’ OR ‘deep vein thrombosis’ OR ‘DVT’ OR ‘pulmonary embolism’ OR ‘PE’) AND (‘arthroscopic hip surgery’ OR ‘hip arthroscopy’). Only English and Chinese publications were included. We additionally searched for relevant clinical guidelines and international consensus statements on HA and VTE. The PRISMA framework guided all search and reporting processes.

### Study screening

Two reviewers independently screened titles and abstracts against eligibility criteria. Articles deemed potentially relevant by either reviewer underwent full-text assessment. Discrepancies during title/abstract screening were resolved by inclusive retention to maximize sensitivity. Full-text screening disagreements were resolved through consensus or, if necessary, adjudication by a senior reviewer. Reference lists and citation tracking were also performed.

### Risk of bias assessment

Methodological quality of included studies was appraised in duplicate using validated tools: the Methodological Index for Non-Randomized Studies (MINORS) for case series and the Newcastle-Ottawa Scale (NOS) for cohort studies (13, 14).

- MINORS: This 12-item instrument assigns scores of 0 (not reported), 1 (reported but inadequate), or 2 (reported and adequate). Maximum scores are 16 for non-comparative and 24 for comparative studies.
- NOS: This 8-item scale evaluates studies across three domains: Selection (4 items), Comparability (1 item), and Outcome/Exposure (3 items). Studies scoring ≥6 points were classified as high quality; scores ≤5 indicated low quality.

### Data extraction

Two reviewers independently extracted data using a standardized form in Microsoft Excel. Extracted variables included: first author, publication year, study design, evidence level (I-V), sample size, patient demographics (age, sex), follow-up duration, preoperative VTE prophylaxis use, traction duration, surgical time, VTE diagnostic methods, incidence rates (DVT, PE, overall VTE), and reported VTE risk factors (e.g., obesity, oral contraceptive use).

### Statistical analysis

The occurrence rates of VTE, DVT, and PE in the included studies were all dichotomous data, which was extracted in the form as an absolute number and patient number. The Mantel-Haenszel (M-H) method was used Heterogeneity was quantitatively assessed using I^2^. Descriptive statistics were calculated. Continuous variables are expressed as weighted means ± standard deviations (SD). Categorical data are presented as frequencies and percentages. Statistical significance was defined as *p* < 0.05 for all analyses; reported *p*-values were either directly extracted or calculated from study data. An intraclass correlation coefficient (ICC) was used to evaluate the inter-reviewer agreement of the MINORS/NOS scores. The ICC value ranges from 0 to 1, with values closer to 1 indicating higher consistency.

## Results

### Literature screening

**Figure 1:**
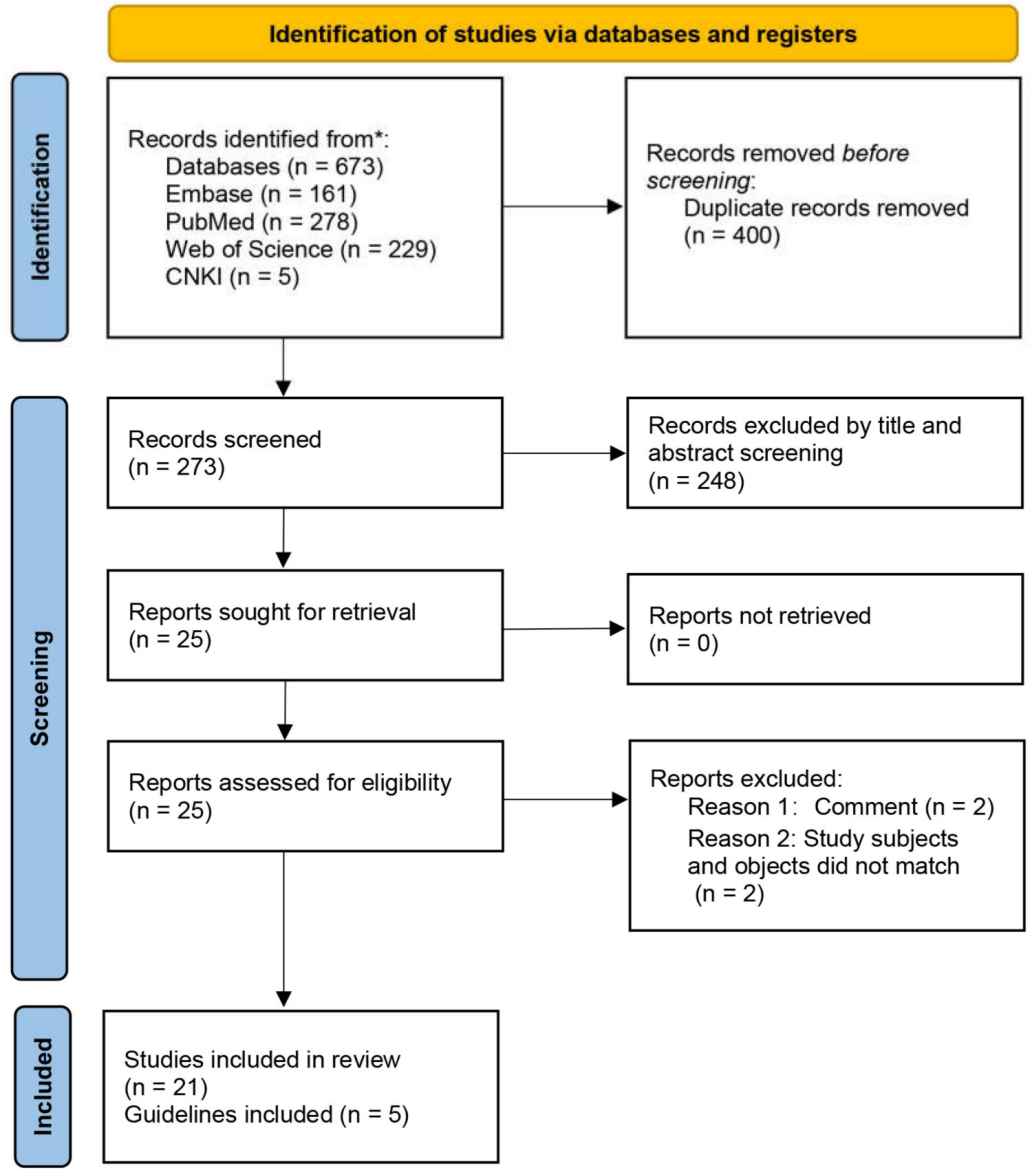
Flow chart of literature screening. CNKI, China National Knowledge Infrastructure.

The initial database search yielded 673 records (Figure 1). After duplicate removal and independent title/abstract screening by two reviewers, 21 articles proceeded to full-text review and met inclusion criteria. The literature comprised: retrospective studies (*n* = 14), prospective investigations (*n* = 2), meta-analyses (*n* = 1), case reports (*n* = 1), and clinical guidelines (*n* = 3) (Tables 1, 2). This systematic process ensured the inclusion of relevant, high-quality evidence.

**Table 1:** The characteristics of the included studies.

References	*n*	Mean follow-up	Risk factors identified	Thromboproph-ylaxis strategy	VTE incidence (%)		
					DVT	PE	VTE
Alaia MJ, et al.(2014) (31)	139	16.12d	Overweight, use of OCP	No	0.7	0.7	1.4
Fukushima K, et al.(2016) (32)	72	7d	/	No	6.94	0	6.94
Schüttler KF, et al.(2018) (33)	485	6w	/	Yes (LMWH, during the period of limited weight)	0	0	0
Cvetanovich GL, et al.2016) (34)	1,338	30d	/	No	0.1	0	0.1
Truntzer JN, et al.(2017) (35)	2,581	1year	/	No	/	/	/
Khazi ZM, et al.(2019) (15)	9,477	90d	Age more than 45 yr, obesity, tobacco use, diabetes, and COPD	No	0	0.43	0.43
Sherman WF, et al.(2022) (36)	19,735	3m	/	No	/	/	/
Holler JT, et al.(2023) (37)	60,181	90d	Use of OCP, obesity, a history of malignancy	/	0.5	0.2	0.7
Gillinov SM, et al.(2023) (8)	31,623	90d	/	/	0.52	0.23	0.75
Malviya A, et al.(2015) (7)	6,395	3m	/	/	0.08	0.08	0.16
Salvo JP, et al.(2010) (38)	81	2w	Use of OCP	No	3.7	0	3.7
Souza BG, et al.(2010) (39)	194	2yr	/	No	0.52	0	0.52
Polat G, et al.(2013) (40)	42	28.2m	/	Yes (not specified)	0	0	0
Kevin Chan, et al.(2013) (41)	236	394 ± 216.5d	/	/	0.8	0	0.8
A. Javed, et al.(2011) (42)	40	30m	/	/	0	0	0
Roos B D et al.(2015) (43)	40	29.1m	/	/	2.43	0	2.43
Beutel BG, et al.(2015) (44)	18	29.4m	/	Yes (Aspirin 325 mg, daily for 2weeks)	0	0	0
Mohtadi NG, et al.(2016) (45)	115	10–22d	/	No	4.3	0	4.3
Larson CM, et al.(2016) (46)	1,615	36.7m	Clotting cascade disorder, arteriovenous anomaly	Yes (Compression stockings and early mobilization)	0.1	0.1	0.2
Collins JA, et al.(2015) (16)	39	2yr	Obesity	Yes (acetylsalicylic acid 325 mg daily for 2 weeks)	5	0	5
Domb BG, et al.(2016) (47)	931	28.8m	/	Yes (Aspirin 325 mg, twice daily for 2 weeks)	0.54	0.21	0.75

Not all studies denoted PE/DVT incidence. yr, year. min, minute. d, day. w, week. m, month. OCP, oral contraceptive pill. COPD, chronic obstructive pulmonary disease./, not reported.

**Table 2:** The characteristics of the included studies.

References	Level of evidence	Study design	MINORS/NOS	Female patients	Mean age	Main arthroscopic procedures	Operation time	Traction duration (min)
Alaia MJ, et al.(2014) (31)	III	Retrospective cohort	7.5	85	37.7 ± 12.0	/	101.6	58.9 ± 23.1
Fukushima K, et al.(2016) (32)	III	Prospective cohort	9	43	46.3 ± 1.7	Cam resection and labral repair	91.2	/
Schüttler KF, et al.(2018) (33)	IV	Case series	8	275	43.9	/	/	77.4 ± 28.7
Cvetanovich GL, et al.2016) (35)	III	Case control	8	798	39.53 ± 12.98	/	/	101.11 ± 53.50
Truntzer JN, et al.(2017) (35)	IV	Case series	9	1,602	/	/	/	/
Khazi ZM, et al.(2019) (15)	III	Retrospective cohort	9	5,085	/	/	/	/
Sherman WF, et al.(2022) (36)	III	Retrospective cohort	7	13,649	/	/	/	/
Holler JT, et al.(2023) (37)	III	Retrospective cohort	9	41,645	/	/	/	/
Gillinov SM, et al.(2023) (8)	IV	Case series	8	22,117	37	Cam resection and labral repair	/	/
Malviya A, et al.(2015) (7)	IV	Case series	9	4,014	38	/	/	/
Salvo JP, et al.(2010) (38)	IV	Case series	9	49	32.2	Labral debridement and femoral osteoplasty	/	/
Souza BG, et al.(2010) (39)	IV	Case series	10	79	36.2	Labial debridement, Osteochondroplasty	/	/
Polat G, et al.(2013) (40)	IV	Case series	9.5	17	35.1	Labial debridement, Osteochondroplasty	/	/
Kevin Chan, et al.(2013) (41)	IV	Case series	11	114	37 ± 13	Labral debridement and femoral osteoplasty	/	/
A. Javed, et al.(2011) (42)	IV	Case series	8	14	65	Pincer resection/acetabuloplasty	/	/
Roos B D et al.(2015) (43)	IV	Case series	8	4	36.12	/	/	/
Beutel BG, et al.(2015) (44)	III	Case control	18	9	56	Pincer resection/acetabuloplasty	54	40
Mohtadi NG, et al.(2016) (45)	II	Prospective cohort	8	57	34.5 ± 10.1	Cam resection, pincer resection, peripheral compartment arthroscopy	/	38
Larson CM, et al.(2016) (46)	IV	Case series	9	805	30.5	Labral repair, cam resection, pincer resection	81	44
Collins JA, et al.(2015) (16)	IV	Case series	8	11	41 ± 10.8	Synovectomy, Chondroplasty, Labral debridement, cam resection, pincer resection	54	48
Domb BG, et al.(2016) (47)	II	Prospective cohort	7.5	541	36.6	Cam resection, pincer resection, Labral repair	/	/

Not all studies denoted main arthroscopic procedures./, not reported.

### Study characteristics and patient demographics

The 21 included studies, published between 2010 and 2025, reported outcomes for 135,377 patients undergoing HA. Evidence levels were: Level II (*n* = 2), Level III (*n* = 7), and Level IV (*n* = 12). Study designs included: cohort studies (*n* = 7) and case series/case-control studies (*n* = 14). Methodological quality assessment yielded a mean MINORS score of 9.5 for case series/control studies and a mean NOS score of 8.1 for cohort studies (Table 2).

### The incidence of VTE after HA

As shown in Supplementary Tables 1, 2; Supplementary Table S1, these studies demonstrated substantial heterogeneity in VTE incidence (range: 0%–6.94%). Meanwhile, it was observed that the incidence rate of VTE in prospective cohort studies was higher than in retrospective cohort studies (Tables 1, 2). When subgroup analysis was conducted according to the study design, the case-control group showed low heterogeneity, with a combined incidence of VTE of 0.07% (Supplementary Table S2; Supplementary Figure S1). Large-scale analyses (*n* > 30,000) reported lower rates (0.7%-0.75%), whereas smaller cohorts (*n* = 18–81) showed higher incidences, suggesting detection bias or underpowered outcomes. Pharmacological prophylaxis (e.g., LMWH, aspirin) reduced VTE risk to very low levels (0%-0.54%), contrasting sharply with no-prophylaxis cohorts (1.4%-5.0%). The incidence rate of pulmonary embolism following hip arthroscopy ranges from 0% to 0.7%.

### Risk factors after HA

The risk factors for VTE following hip arthroscopy have been reported in various studies. As shown in Table 1, multiple studies suggest that obesity and oral contraceptive use are significant risk factors for VTE after HA. One study indicates age ≥45 is also a risk factor for VTE after HA (15). Additionally, smoking, chronic obstructive pulmonary disease (COPD), diabetes mellitus, history of malignancy, and vascular malformations have also been identified as risk factors for VTE after HA.

### Thromboprophylaxis recommendations after HA: different guidelines

Currently, no dedicated guidelines exist for thromboprophylaxis following HA. Recommendations for HA are addressed only in brief within select general orthopedic thromboprophylaxis guidelines. Table 3 summarizes the part of the guidelines for the prevention of venous thromboembolism that mentions hip arthroscopy. These recommendations collectively advocate for risk-adapted prophylaxis, though variations in implementation reflect differing clinical priorities. The American College of Chest Physicians (ACCP) emphasizes a risk-stratified approach, reserving pharmacological prophylaxis (e.g., anticoagulants) for high-risk patients with factors such as prior VTE, obesity, or immobility, while recommending physical prophylaxis (e.g., compression devices) for those without additional thrombotic risks. The UK National Institute for Health and Care Excellence (NICE) employs the Caprini score to guide prophylaxis: low-to-moderate risk patients receive mechanical prophylaxis combined with early mobilization, whereas high-risk cases are advised to incorporate low-molecular-weight heparin (LMWH).

**Table 3:** Guidelines for VTE that mentioned HA.

Organization	Recommendation
The American College of Chest Physicians: Antithrombotic Therapy and Prevention of Thrombosis, 9th ed (2012) (48)	For non-major surgeries without additional thrombotic risks, physical prevention is recommended. If high-risk factors are present (such as a history of VTE, obesity, or immobility), pharmacological prevention may be considered.
The UK National Institute for Health and Care Excellence, ng89 (2018) (49)	Based on the Caprini score, mechanical prevention combined with early mobilization is recommended for low-to-moderate risk patients. For high-risk patients, pharmacological prevention (such as low molecular weight heparin) is advised.
The German Association of Scientific Medical Societies (2023) (50)	Routine anticoagulation is not recommended unless the patient has a history of VTE or is immobile. Mechanical prevention and early postoperative mobilization are advised.
The Expert Committee on the Guidelines for the Prevention and Treatment of Thrombotic Diseases in China (2018) (51)	Routine VTE prophylaxis is not recommended for arthroscopic surgery patients, but high-risk cases should receive pharmacological prophylaxis.
International Consensus Meeting Recommendations (2023) (52)	Routine administration of VTE prophylaxis to patients undergoing HA is not supported. However, patients at higher risk of VTE may benefit from the use of mechanical and/or pharmacological prophylaxis.

The German Association of Scientific Medical Societies (AWMF) discourages routine anticoagulation unless patients exhibit a history of VTE or immobility, prioritizing mechanical methods as first-line interventions. The Chinese Expert Committee on Thrombotic Diseases opposes universal prophylaxis for HA patients but endorses pharmacological measures for high-risk subgroups.

A recent International Consensus Meeting (ICM), involving over 600 experts from 68 countries and 135 societies. The ICM recommends that routine administration of VTE prophylaxis to patients undergoing HA is not supported by the current data. However, patients at higher risk of VTE may benefit from the use of mechanical and/or pharmacological prophylaxis, which may include aspirin.

## Discussion

### VTE incidence after HA

Venous thromboembolism (VTE), comprising deep vein thrombosis and pulmonary embolism, carries a mortality risk when not managed. Thus, VTE constitutes a serious complication following hip arthroscopy (HA). This systematic review identifies a post-HA VTE incidence ranging from 0% to 6.94%, while pulmonary embolism specifically occurs in 0%–0.7% of cases. Collins et al. reported patients undergoing pharmacological prophylaxis exhibited a higher incidence of VTE. However, this finding was derived from a study with a limited sample size of 39 participants (16). Pooled data from these studies show marked variability in VTE incidence, potentially reflecting underreporting or inconsistent prophylaxis adherence. The observed heterogeneity in VTE incidence after hip arthroscopy arises from multiple interrelated factors. First, study design and detection bias are critical factors. Prospective cohorts using active screening methods (e.g., ultrasound) report significantly higher DVT rates, ranging from 4.3% to 6.94%. In contrast, retrospective studies relying on symptomatic diagnosis show lower rates of 0.08% to 3.7%. This disparity underscores how surveillance intensity affects outcome reporting. Prophylaxis practices also influenced variability. Pharmacological interventions (e.g., aspirin, LMWH) reduced VTE risk by over 90%, outperforming mechanical or no prophylaxis. This underscores the need for standardized preventive protocols. Additionally, surgical complexity increased risks-especially prolonged traction (>50 min) and bony resection procedures. However, inconsistent reporting of operative details hindered cross-study comparisons.

### Risk factors

This review establishes obesity, oral contraceptive use, age ≥45 years, smoking, chronic obstructive pulmonary disease, diabetes mellitus, history of malignancy, and vascular malformations as significant VTE risk factors post-HA. Oral contraceptive use enhances thrombin production, evidenced by elevated D-dimer and prothrombin fragment (F1 + 2) levels. Concurrently, hormone therapy modulates endothelial activity. Emerging evidence indicates that estrogen dosage correlates with matrix metalloproteinase expression, triggering degradation of vascular collagen/elastin networks. This cascade promotes venous stasis, heightened vascular permeability, and ultimately predisposes to thrombus formation (12). However, a meta-analysis revealed that obesity, smoking, and age >45 years are significant risk factors for postoperative VTE following hip arthroscopy (17). This discrepancy might be due to variations in the included studies, as this meta-analysis only incorporated five studies. In contrast, factors like obesity, COPD, history of malignant tumor, and vascular abnormalities have been identified as VTE risk factors in multiple studies and are integrated into the Caprini risk assessment score (18, 19). Among 21 included studies, six implemented pharmacological thromboprophylaxis, with three reporting zero VTE events and two documenting low incidences (0.2% and 0.75%). Notably, Collins et al. observed 5% VTE incidence despite prophylaxis-a finding attributable to their exclusive focus on obese patients (BMI ≥ 30), a high-risk subgroup. Obesity is a recognized risk factor for venous thromboembolism. Obesity may cause thrombosis due to the activity of adipocytokines, such as leptin and adiponectin, increasing coagulation activity and inflammation and decreasing the fibrinolytic cascade (20). Prolonged surgical and traction durations appear associated with elevated VTE risk, as orthopedic traction generates tourniquet-like effects. Simultaneously, anesthesia-induced calf muscle paralysis promotes venous stasis. Although surgeons routinely monitor traction duration, the cumulative anesthesia duration (including postoperative recovery) remains clinically overlooked.

### VTE risk assessment tools

Numerous risk assessment tools have been developed for thromboembolism evaluation, including the Caprini Risk Assessment Model (RAM), Rogers Score, and Padua Prediction Score (18, 19, 21, 22). The Padua Prediction Score is primarily employed for medical inpatients, while the Caprini RAM finds extensive application in surgical populations. Unlike the Caprini RAM, the Rogers Score omits consideration of certain critical VTE risk factors, notably personal or familial history of VTE and inherited thrombophilia. However, there remains a paucity of evidence regarding the optimal risk assessment tool for patients undergoing HA. The Caprini Risk Assessment Model was originally developed for general and vascular surgery patients. It provides a systematic approach to quantify thrombotic risk. Based on calculated risk scores, clinicians can formulate corresponding prophylactic strategies. Notably, the Caprini scoring system has demonstrated successful clinical adaptation and validation in plastic surgery (23–27), suggesting potential applicability for direct implementation in HA populations. Crucially, no existing risk assessment instrument has undergone rigorous clinical validation specifically for HA procedures. When using scoring systems for postoperative thromboprophylaxis decisions in this surgical setting, clinicians should practice careful interpretation. They must integrate both quantitative risk stratification and patient-specific considerations. A comprehensive patient-specific considerations is essential.

### Guidelines and recommendations

Current global thromboprophylaxis guidelines advocate for personalized approaches, yet substantial regional disparities persist in anticoagulant utilization patterns (28). Notably, no consensus exists regarding optimal pharmacologic prophylaxis following orthopedic procedures. Emerging evidence suggests that in non-major orthopedic surgery, the risks of VTE and bleeding complications remain comparable between rivaroxaban and LMWH regimens (29). Regarding total knee arthroplasty (TKA), a growing body of research indicates that aspirin demonstrates inferior efficacy to LMWH in VTE prevention, with studies reporting significantly higher postoperative thrombosis rates with aspirin monotherapy (30). This finding may partially explain why European and Asian clinical frameworks exercise greater restraint in routine pharmacological prophylaxis, prioritizing bleeding risk mitigation over universal anticoagulation. Moving forward, the standardization of risk stratification protocols and the development of procedure-specific outcome data will be critical to establishing globally harmonized clinical practices.

### Limitations

This study is constrained by methodological limitations inherent to the extant low-quality evidence base. Among the 21 included studies, case-control or case series accounted for 14 (predominantly Level IV evidence), which may compromise the reliability of the conclusions. Substantial discrepancies in reported surgical parameters (anesthesia duration, traction time, and postoperative follow-up intervals) across studies in both VTE prophylaxis regimens and detection protocols, significantly complicate the formulation of definitive clinical recommendations. Furthermore, while acknowledging the inherent difficulty in disaggregating hip-specific outcomes from general patient data within published literature, such confounding variables exerted negligible impact on pooled rates.

## Conclusions

Current evidence indicates a clinically significant discrepancy between symptomatic (low incidence) and imaging-detected (up to 6.9%) VTE rates after hip arthroscopy, underscoring the necessity of protocolized screening for accurate risk stratification. Thus, the distinction between symptomatic and imaging-detected VTE is critical for risk stratification. While routine prophylaxis may not be necessary for all patients, omitting preventive measures should be based on thorough risk assessment-especially given the high rate of imaging-detected VTEs that often go unnoticed. High-risk patients (e.g., those with obesity, oral contraceptive use, age ≥45 years) should receive tailored thromboprophylaxis, including pharmacological prophylaxis when appropriate. To clarify current uncertainties, future studies should focus on standardized screening methods and risk models specific to hip arthroscopy. Until stronger evidence is available, doctors must weigh each patient's unique risks, hospital guidelines, and the shortcomings of symptom-based monitoring to prevent VTE effectively while avoiding unnecessary anticoagulation side effects.

## Data Availability

The original contributions presented in the study are included in the article/supplementary material, further inquiries can be directed to the corresponding author.
