# Supplementary material for: Venous thromboembolism after hip arthroscopy: a systematic review of incidence, risk factors, and international guidelines
Source: Front Surg. 2025 Oct 14;12:1658428. doi: 10.3389/fsurg.2025.1658428 (PMC12558938; doi:10.3389/fsurg.2025.1658428)
Supplement: Supplementary file 1 [file Supplementaryfile1.docx]

| Supplementary Table1. Heterogeneity of all included studies | | | | | | | |
| --- | --- | --- | --- | --- | --- | --- | --- |
| Thrombosis | No. of included studies | Total | Events | Incidence | 95% CI | MH I^2^ | P value |
| VTE | 21 | 135,377 | 741 | 0.19% | (0.08-0.34) | 97.20% | <0.001 |
| DVT | 21 | 135,377 | 498 | 0.16% | (0.06-0.3) | 95.90% | <0.001 |
| PE | 21 | 135,377 | 244 | 0.03% | (0.02-0.06) | 91.20% | <0.001 |

| Supplementary Table2. Subgroup heterogeneity analysis according to study design | | | | |
| --- | --- | --- | --- | --- |
| Study design | No. of included studies | Events | Combined incidence | MH I^2^ |
| Case control | 2 | 1/1356 | 0.07% | 0.00% |
| Case series | 12 | 259/43371 | 0.17% | 94% |
| Prospective cohort | 3 | 17/1118 | 0.88% | 72.00% |
| Retrospective cohort | 4 | 464/89532 | 0.01% | 99% |


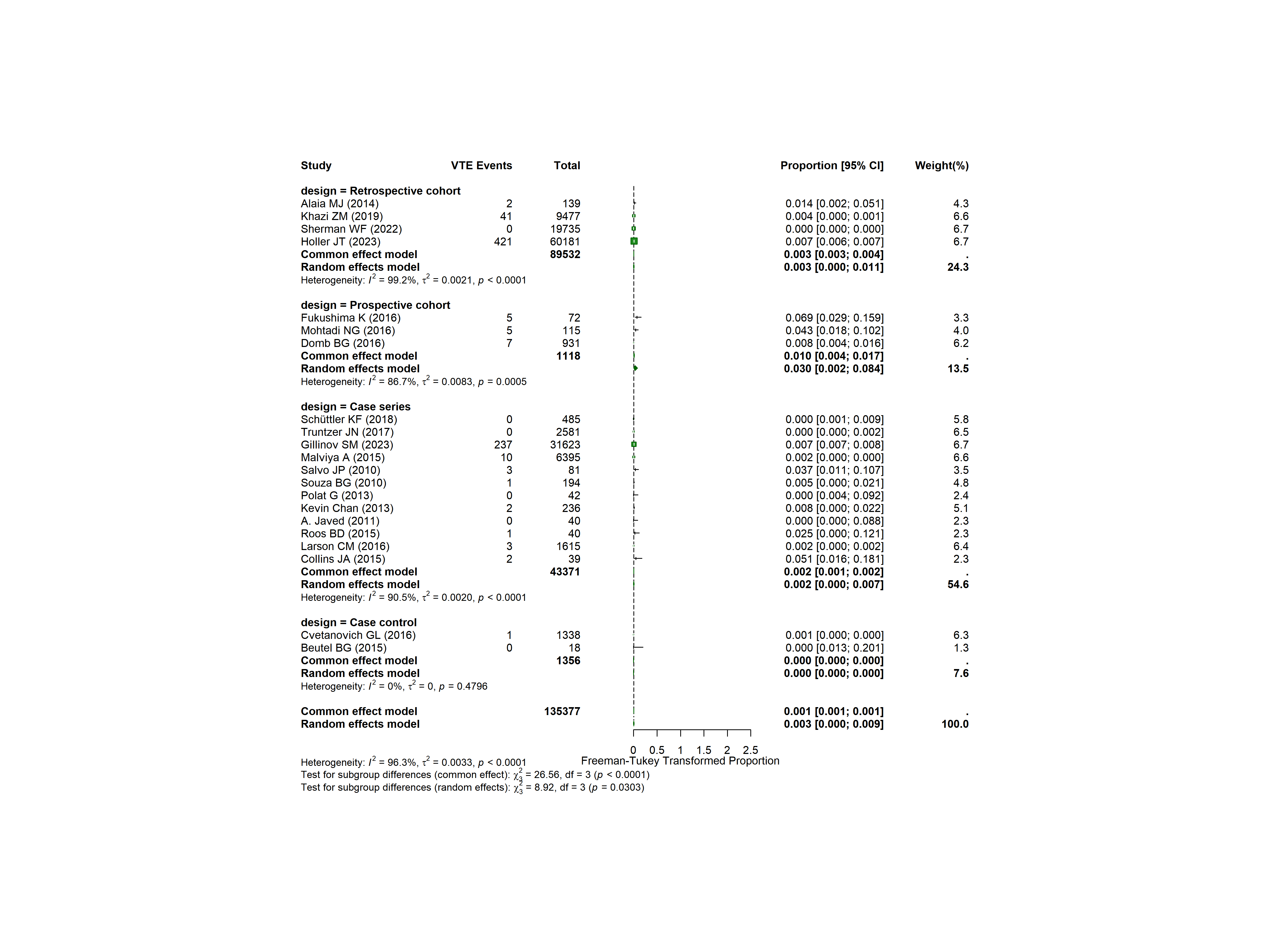


Supplementary Figure1. The pooled VTE events

**Search strategy**

PubMed

arthroscop* AND ("Hip"[Mesh])

Embase

'hip arthroscopy'/exp AND 'venous thromboembolism'/exp

WOS

Hip AND arthroscopy AND (Venous Thrombosis OR Phlebothrombosis OR Phlebothromboses OR Thrombosis, Venous OR Thromboses, Venous OR Venous Thromboses OR Deep Vein Thrombosis OR Deep Vein Thromboses OR Vein Thrombosis, Deep OR Deep-Venous Thrombosis OR Deep-Venous Thromboses OR Thromboses, Deep-Venous OR Thrombosis, Deep-Venous OR Deep-Vein Thrombosis OR Deep-Vein Thromboses OR Thrombosis, Deep-Vein OR Thrombosis, Deep Vein OR Deep Venous Thrombosis OR Deep Venous Thromboses OR Thromboses, Deep Venous OR Thrombosis, Deep Venous OR Venous Thromboses, Deep OR Venous Thrombosis, Deep OR DVT OR Pulmonary Embolism OR Pulmonary Embolism OR Pulmonary Embolisms OR Embolism, Pulmonary OR Embolisms, Pulmonary OR Pulmonary Thromboembolisms OR Pulmonary Thromboembolism OR Thromboembolism, Pulmonary OR Thromboembolisms, Pulmonary OR PE)

CNKI

髋关节镜 AND 静脉血栓
